# Danger from Hypodermic Injection of Morphia

**Published:** 1878-05

**Authors:** E. Fletcher Ingals

**Affiliations:** Chicago


### DANGER FROM THE HYPODERMIC INJECTION OF MORPHIA.

By E. Fletcher Ingals, M. D., Chicago.

In the August number of this journal, in connection with three cases reported by Dr. E. Wenger, of Gilman, Ill., I presented the history of a case illustrative of the danger which sometimes attends the hypodermic injection of morphia.

In reply to this article, a communication from Dr. John McLean, of Du Quoin, Ill., appeared in the November number, which induced me to make further inquiry upon the subject. Accordingly, I addressed circulars embracing the following questions, to eighty representative physicians of the northwest:

"Have you often given hypodermic injections of morphia?"

"What is your ordinary dose of morphia when used in this manner?"

"Have you ever seen syncope or other unpleasant results follow its administration by this method?"

I have received replies to fifty-five of these circulars. Nearly all of my correspondents had used hypodermic injections often, in doses varying from $\frac{1}{16}$ to $\frac{1}{2}$ of a grain, and in exceptional cases one grain. The majority were accustomed to give from $\frac{1}{6}$ to $\frac{1}{4}$ or even $\frac{1}{2}$ of a grain. Two recommended from $\frac{1}{16}$ to $\frac{1}{2}$ of a grain. Three did not exceed $\frac{1}{6}$ of a grain.

A few stated that they were accustomed to combine atropia with morphia for hypodermic use. Thirty-four, of the fifty-five, had seen no unpleasant results, and six had seen nothing worse than abcesses at the point of introduction.

The unpleasant results observed by the remaining fifteen are well worthy of our attention, and ought to lead us to greater caution in the use of morphia in this manner. When this drug has been taken into the stomach, there is still a possibility of removing a portion of it, and doubtless, as the system comes gradually under its influence, absorption is more and more impeded, so that the danger from any which may remain in the stomach, is constantly diminishing, and time is allowed for treatment adapted to such cases. If morphia is introduced by the hypodermic syringe, it passes speedily, if not directly into the circulation, and may overwhelm the nervous system by toxic effects, which the physician will have neither time nor means to counteract. It is not unlikely that the most unfortunate experiences were among those who made no reply to my inquiries; still I have received reports of seven fatal cases from morphia used subcutaneously. Had the same dose been given internally, there is reason to believe that six of these would not have occurred, and possibly the seventh might have been saved by proper treatment.

I have no desire to limit the use of a remedy which does so much to alleviate human suffering; neither do I care to discourage its subcutaneous administration, provided it can be so used with safety; but I wish to point out the danger of serious effects, to those who from long use without accident, have come to believe the hypodermic injection harmless, and to those who have not thought of its dangers.

I have no doubt that a minute dose of morphia would be harmless, even if injected directly into a blood vessel; but I fear it would have little effect in relieving severe pain, unless frequently repeated. Even the $\frac{1}{12}$ of a grain is not always devoid of danger, and a fatal case from $\frac{1}{5}$ of a grain of morphia, combined with one $\frac{1}{75}$ of a grain of atropia, shows that it is not safe to rely on the antagonistic effects of the latter.

The results of my inquiries seem to leave us no other choice than reasonable doses by the stomach, or the frequent repetition of *very small doses*, hypodermically. The method is of little importance, provided it is safe and agreeable to the patient.

In two of the seven fatal cases reported, the amount of morphia administered was not ascertained, though it was believed not to have exceeded the ordinary dose. In the other cases, the doses and the condition of the patients were given, but I am not at liberty to report the cases fully; however, as my information was derived from men of large experience and recognized ability, I can vouch for its accuracy. The names of my correspondents are withheld for obvious reasons.

One reports two deaths, one of which was caused by the subcutaneous injection of $\frac{1}{5}$ of a grain of morphia, combined with $\frac{1}{75}$ of a grain of atropia.

Another reports two deaths, one from two doses of morphia, of $\frac{1}{3}$ of a grain each, with an interval of four hours between the first and second doses. In this instance the morphia was given to relieve the intense pain attending invagination of the intestines. Death from narcotism ensued six hours after the second dose. The other death reported by this physician, resulted from $\frac{1}{4}$ of a grain given in a case of sciatica. The patient died comatose within five hours.

Another reports a death caused by two doses of $\frac{1}{4}$ of a grain each; the first given internally.

The sixth case was that of a patient upon whom ovariotomy had been performed. Shortly after the operation, this patient was given subcutaneously, ten minims of a saturated solution of morphia, which amount was repeated five hours later on account of pain; in fifteen or twenty minutes the latter dose was followed by profound narcotism, and within two hours death ensued, notwithstanding persistent use of the most approved treatment. In this case the exact dose was not known, but, considering the ready solubility of morphia, it probably equalled, and may have exceeded $1\frac{1}{2}$ grains. The physician who gave me the notes of this case, found that ten minims of water at 60° F., would dissolve $1\frac{1}{2}$ grains of morphia, while at 160° F., it would dissolve $6\frac{1}{2}$ grains.

The seventh case I give as reported to me:

"I was called to see Mr. B., who lived seven miles away, at 8 o'clock p. m., and I found him suffering from myalgia of the muscles of the back. I at once administered one-sixtieth ($\frac{1}{60}$) of a grain of sulphate of atropia hypodermically; after waiting till dryness of the throat and other constitutional symptoms showed themselves, and finding it gave him no relief from pain, I gave him $\frac{1}{4}$ of a grain of morphia by the mouth. In three quarters of an hour from the time I gave the morphia, the man was still groaning with pain, and then I gave $\frac{1}{4}$ of a grain of morphia hypodermically, which soon quieted the patient. Not suspecting any unpleasant effects from the medicine, I took my departure. In a couple of hours I was sent for to see the man, who, the messenger stated, 'was in a deep stupor.' Upon my arrival I found Drs. L—— and J——, who lived only three miles distant. The patient's breathing was stertorous, and there was complete narcotism. Dr. J—— stated that the pupils were not contracted when he arrived. He had given atropia hypodermically, and was using electricity. The latter was kept up as well as artificial respiration, till the patient died, which was about twelve hours from the time the morphia was given. I have given morphia hypodermically a great many times, and repeated $\frac{1}{4}$ grain doses as often as every twenty minutes, but never before saw any such effect as was produced in this case."

In order better to illustrate some of the effects which follow the hypodermic injection of morphia, I quote from the replies to some of my circulars.

"I think there is always more or less danger from the use of morphia in this way, so have almost discarded its use."

"I am chary of the use of the syringe; using it when demanded—dose gr. $\frac{1}{8}$. Largest dose ever ventured on was gr. $\frac{1}{3}$ (in violent colic.) The patient was asleep in one minute, and did not awake for sixteen hours. No bad results."

"I have seen unexpectedly severe effects on several occasions. I do not give morphia in that way, when I can give it by the stomach. The effects that I speak of were simply severe ordinary effects of the medicine."

"No syncope, but in my experience results have been so uncertain and unexpected, that I now never resort to the surgical operation to introduce medicine, when the patient can swallow or has the use of the rectum. I should as soon think of introducing food as a regular thing by transfusion of blood. Put me down as opposed to the practice, save in exceptional cases."

"Once after $\frac{1}{6}$ of a grain, syncope occurred in about fifteen minutes after administration; was given in a case of cholera morbus."

"One case, the patient slept twenty-four hours. * * * The dose was only $\frac{1}{12}$ of a grain."

"But once, gave $\frac{1}{3}$ gr. to adult male (in case of compound comminuted fracture of tibia and fibula), to ease pain. While administering injection, sudden collapse and dyspnœa, with fluttering heart. Rallied under ammonia and whisky." * * *

"In the case of an old lady nearly eighty years of age, there was almost instantly after the injection a frightful degree of numbness, as she expressed it. (The dose gr. $\frac{1}{4}$.)" * * *

"Have seen alarming syncope in three cases, great prostration in a score at least." * * *

I know of no precaution which will render the hypodermic injection of sedative doses of morphia entirely safe ; the medicine may be given in this way a thousand times without harm, but the next time it may be the cause of death. Nothing in the patient's history or general appearance can warn us of the danger, for we find fatal accidents among those who have formerly taken morphia with the happiest results. The danger seems to arise from rapid absorption, or injection directly into the circulation, and it is greatly enhanced by the impossibility of removing the poison.

All are familiar with the rules laid down for the cautious use of the hypodermic syringe, i.e., to withdraw the point of the syringe slightly after the needle has been introduced as far as desired, to inject the solution slowly, etc. ; but these are not sufficient to insure us against accidents.

Withdrawing the point of the syringe will not necessarily prevent the solution from passing directly into the circulation ; and as to injecting slowly, probably no one would think of occupying more than five or, at most, ten minutes in the operation, which would not be long enough to prevent accidents from rapid absorption. So far as danger is concerned, the hypodermic use of morphia seems to stand in much the same relation to its administration by the stomach, as the inhalation of chloroform does to that of ether; either method may be fatal, but the latter seems much the safest.

It is difficult for those who have administered morphia subcutaneously for years, without accident, to realize that there can be danger from the practice; and so is it with chloroform, but we all know that accidents happen from this drug in the hands of the most cautious and skillful surgeons, and considering the large percentage of these fifty-five physicians who have had unpleasant or fatal results from the hypodermic injection of morphia, it would not seem strange if extended and accurate statistics should prove this practice almost if not quite as dangerous as the inhalation of chloroform.

